# Induction and maintenance of bi-functional (IFN-γ ^+^ IL-2^+^ and IL-2^+^ TNF-α^+^) T cell responses by DNA prime MVA boosted subtype C prophylactic vaccine tested in a Phase I trial in India

**DOI:** 10.1371/journal.pone.0213911

**Published:** 2019-03-28

**Authors:** Sivasankaran Munusamy Ponnan, Sathyamurthy Pattabiram, Kannan Thiruvengadam, Rajat Goyal, Nikhil Singla, Joyeeta Mukherjee, Shweta Chatrath, Philip Bergin, Jakub T. Kopycinski, Jill Gilmour, Sriram Kumar, Malathy Muthu, Sudha Subramaniam, Soumya Swaminathan, Srikanth Prasad Tripathy, Hanna Elizabeth Luke

**Affiliations:** 1 Department of HIV, National Institute for Research in Tuberculosis (Indian Council of Medical Research), Chennai, India; 2 International AIDS Vaccine Initiative, New Delhi, India; 3 IAVI Human Immunology Laboratory, Imperial College, London, United Kingdom; George Washington University School of Medicine and Health Sciences, UNITED STATES

## Abstract

Effective vaccine design relies on accurate knowledge of protection against a pathogen, so as to be able to induce relevant and effective protective responses against it. An ideal Human Immunodeficiency virus (HIV) vaccine should induce humoral as well as cellular immune responses to prevent initial infection of host cells or limit early events of viral dissemination. A Phase I HIV-1 prophylactic vaccine trial sponsored by the International AIDS Vaccine Initiative (IAVI) was conducted in India in 2009.The trial tested a HIV-1 subtype C vaccine in a prime-boost regimen, comprising of a DNA prime (ADVAX) and Modified Vaccine Ankara (MVA) (TBC-M4) boost. The trial reported that the vaccine regimen was safe, well tolerated, and resulted in enhancement of HIV-specific immune responses. However, preliminary immunological studies were limited to vaccine-induced IFN-γ responses against the Env and Gag peptides. The present study is a retrospective study to characterize in detail the nature of the vaccine-induced cell mediated immune responses among volunteers, using Peripheral Blood Mononuclear Cells (PBMC) that were archived during the trial. ELISpot was used to measure IFN-γ responses and polyfunctional T cells were analyzed by intracellular multicolor flow cytometry. It was observed that DNA priming and MVA boosting induced Env and Gag specific bi-functional and multi-functional CD4^+^ and CD8^+^ T cells expressing IFN-γ, TNF-α and IL-2. The heterologous prime-boost regimen appeared to be slightly superior to the homologous prime-boost regimen in inducing favorable cell mediated immune responses. These results suggest that an in-depth analysis of vaccine-induced cellular immune response can aid in the identification of correlates of an effective immunogenic response, and inform future design of HIV vaccines.

## Introduction

HIV vaccine research aims to prevent infection or reduce viral load and thereby slow down disease progression [1]. Lack of natural protective immunity against HIV is the main hindrance to the development of a protective vaccine. This suggests that an effective candidate vaccine that elicits immune responses that are superior to the natural immune response will be required to protect against HIV infection [2]. Other challenges include the high degree of viral genetic variation, lack of ideal animal models, and functional limitations in performing large-scale clinical trials [3–4]. Some DNA constructs have been demonstrated to be effective in moderately reducing the viral load in macaques infected with Simian Immunodeficiency Virus (SIV) or Simian/Human Immunodeficiency Virus (SHIV) [5]. Vector-based heterologous immunizations have been instrumental in elevating the breadth and magnitude of vaccine specific immune responses, through initial priming and successive boosting with similar DNA constructs [6]. Researchers believe that there is an urgent need for vaccine candidates that can constitutively induce broadly neutralizing antibodies and a strong cell-mediated response. Hence, the new approach on vaccine development focuses on a prime-boost strategy with a DNA or vector vaccine to elicit cytotoxic T cells that destroy infected cells followed by a subunit vaccine to induce neutralizing antibodies. These heterologous immunizations are useful in stimulating the complementary entities of the immune system to synergistically act against the immunogen [7].

Antigen-specific T cell responses against intracellular pathogens have been commonly characterized based on IFN-γ production [8]. Besides IFN-γ, antigen-specific T cells have also been reported to produce other cytokines like tumor necrosis factor-α (TNF-α) and interleukin-2 (IL-2) following infection and/or vaccination. Induction of polyfunctional and bi-functional memory cells and neutralizing antibodies are desirable vaccine-induced responses [9]. Long-term T cell-mediated protection requires the induction of memory cells to protect against future pathogen challenge. The magnitude of the CD4^+^ or CD8^+^ T cell cytokine response can be worked out effectively by enumerating T cells co-producing IFN-γ, IL-2 and TNF-α, and may be considered a better correlate of vaccine-induced protection as compared to IFN-γ alone [10]. CD4^+^ T cells are critical for the induction and maintenance of CD8^+^ T cell and B cell responses. The main contribution of CD4^+^ T cells is in the generation and differentiation of CD8^+^ cytotoxic T cell responses (CTL) required for controlling viral replication [10, 11], and in the mobilization of CTLs to peripheral sites of infection [12]. HIV-infected individuals with good numbers of antigen-specific CD8^+^ T cells that simultaneously produce multiple cytokines have been shown to have lower viral loads as compared to individuals producing fewer cytokines [13–16]. A positive correlation was observed between slow disease progression and polyfunctionality of HIV-specific CD8^+^T-cells that were selectively characterized in terms of degranulation, production of cytokines (IFN-γ, TNF-α, and IL-2) and chemokine’s (MIP-1β).This suggests that functionally variable antigen-specific CD8^+^ T-cell responses help in protecting against HIV disease progression [17]. Similarly, polyfunctional vaccine-specific CD4^+^ T cells have also been assessed in HIV vaccinated individuals, and found that in addition to IFN-γ, vaccination also induced TNF-α, MIP-1β and IL-2 secretion [18]. CD4^+^T cells promote B cell differentiation into plasma cells to produce neutralizing antibodies and assist memory B cells during re-infection. Thus, CD4^+^ T cells play a central role in memory T and B cell development [19].

HIV vaccines based on plasmid DNA and/or live recombinant virus vectors have been shown to predominantly elicit T cell responses that can control virus replication and delay or prevent CD4^+^ T cell decrease. A recent study in non-human primates showed that macaques vaccinated with a CMV vector expressing the full SIVMAC239 genome elicited robust T Effector Memory (T_EM_) responses, and that animals with polyfunctional T_EM_ cells were less likely to become infected following low dose challenge [20]. Similar T cell responses against conserved CD4^+^T cell epitopes were observed in BALB/c mice vaccinated with a DNA vaccine encoding 18 conserved multiple HLA-DR-binding HIV-1 CD4 epitopes (HIVBr18)[21]. A recent phase II study with the F4/AS01_B_ candidate vaccine (NCT00434512) showed that vaccination of healthy HIV-uninfected volunteers with an adjuvanted polyprotein induced significant numbers of CD4^+^ T cells co-expressing IL-2, IFN-γ and TNF-α. Similar CD4^+^ T cell responses against p17, p24, RT and Nef antigens were also observed in natural viral controllers, suggesting that it is possible to have a candidate vaccine-adjuvant polyprotein that could induce a similar immune response as observed in HIV-infected persons who spontaneously control the virus [22]. The EuroVacc 02 phase I trial with recombinant DNA and the poxvirus vector NYVAC, both expressing a common immunogen consisting of Env, Gag, Pol and Nef polypeptide from a HIV-1 clade C isolate, induced T cell responses predominantly against the Env antigen with polyfunctionality seen in both the CD4^+^ and CD8^+^ T cell subsets [23].

A phase I/II randomized placebo-controlled trial in Tanzania, that tested a HIV-1 DNA prime and HIV-1 MVA boost reported that broader cell-mediated immune responses were observed against the Env after vaccination [24]. Another study that tested the administration of 3 doses of the Geovax MVA/HIV62, vaccine, demonstrated induction of T cell responses with IFN-γ or IL-2 production as assessed by ICS with a strong preference for Gag reactivity. On the other hand, antibody response rates were lower even after two HIV DNA prime immunizations and two MVA/HIV62 boosts [25].

The safety and immunogenicity of a heterologous regimen involving two vector-based HIV-1 subtype C vaccines, ADVAX as DNA prime and MVA TBC-M4 as boost, were evaluated in a phase-I trial in London. The trial revealed that stronger vaccinia-specific cellular responses were elicited in vaccinees immunized with the heterologous regimen as compared to those immunized only with TBC-M4 (homologous regimen). The study concluded that although the ADVAX-prime MVA-boost heterologous regimen resulted in an effective HIV-specific cellular response, it was unsuccessful in subsequently eliciting a competent humoral response [26]. A similar phase I trial conducted in India with the same vaccine constructs revealed that the homologous regimen elicited stronger humoral responses as compared to the heterologous regimen, whereas both regimens elicited similar IFN-γ responses in the volunteers [27]. However, earlier studies did not evaluate the induction and distribution of polyfunctional vaccine specific T cells and other memory subsets. The current study describes the detailed characterization of HIV-specific cellular responses elicited by the heterologous and homologous vaccine regimens with ADVAX and or TBC-M4, utilizing archived PBMC samples of the trial participants that were stored during the conduct of the trial at one of the Indian trial sites, the National Institute for Research in Tuberculosis (NIRT) [27].

## Materials and methods

### Study samples

**Fig 1:**
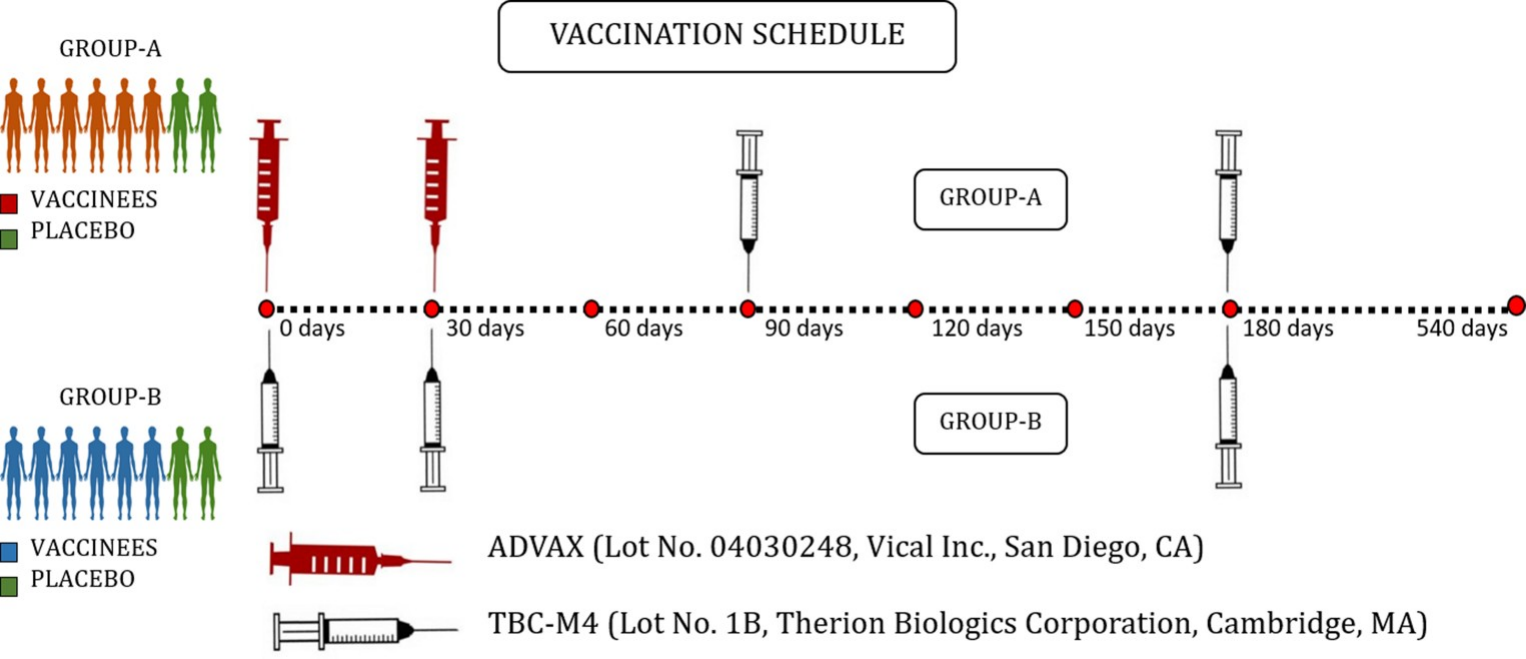
Vaccination schedule of IAVI Phase-I Prime Boost HIV-1 Subtype-C Prophylactic Vaccine Trial-NIRT-ICMR (P001 Trial).

As stated above, the trial was conducted at two sites (NIRT, Chennai and NARI, Pune) and enrolled 16 HIV-uninfected healthy volunteers from April 2009 to December 2010 at each study site [27]. The 16 volunteers enrolled at NIRT (9 males and 7 females) were randomly assigned to either group A or B, with eight participants in each group. Group A participants received two intramuscular (I.M.) injections of ADVAX or placebo in the upper arm at baseline (time ‘0’) and 1 month, followed by two I.M. injections of TBC-M4 or placebo at months 3 and 6. Group B participants received three I.M. injections of TBC-M4 or placebo at time 0, months 1 and 6. Among the 8 volunteers in each group, 6 received the vaccine and 2 received placebo (Fig 1) and all the analysis reported in this study was performed on Cryopreserved peripheral blood mononuclear cells (PBMCs) during the period 2015–2016.

### Candidate vaccines

ADVAX is a DNA-based HIV-vaccine formulated by the Aaron Diamond AIDS Research Center (ADARC), New York, USA, and manufactured by Vical Inc., San Diego, CA, USA (Lot# 04030248), utilizing the structural features of the commercial plasmid backbone, pVAX1 [28].The vaccine contained two plasmid constructs mixed in a 1:1 ratio of one plasmid cloned with the coding sequences of *gag* and *env* genes of the Chinese HIV-1 clade-B/C strain and the other cloned with the coding sequences of nef/tat and pol. The candidate vaccine was suspended in sterile phosphate-buffered saline with 0.01 M sodium phosphate and 150 mM sodium chloride, and was formulated to contain 4 mg of the HIV-peptides in a total volume of 1 mL.

TBC-M4 is another DNA-based HIV-vaccine manufactured by Therion Biologics Corporation, Cambridge, MA, USA (Lot# 1B), It is a recombinant Modified Vaccinia Ankara (MVA) virus containing the coding sequences of gag, env, reverse transcriptase (RT), tat, rev and nef of the Indian HIV-1 clade C strain. The candidate vaccine was suspended in phosphate buffered saline with 10% glycerol so as to contain 5x10^6^ PFU in a total inject volume of 0.5 mL. The amino acid sequence homology between the two vectors was found to be greater than 85% for most of the proteins (env: 87.1%; gag: 95%; pol/RT: 96.4%).

### Ethics statement

The present study was scrutinized and approved by the Institutional Ethics Committee (IEC) of National Institute for Research in Tuberculosis (NIRT) (NIRT IEC N0-2015013). During the trial, written statements of informed consent were obtained from the study volunteers. The trial was supervised by the research personnel of International AIDS Vaccine Initiative (IAVI) and was carried out following the ethical principles stated in the Declaration of Helsinki (DoH), Guidelines for Good Clinical Practice (GCP) framed in the International Conference on Harmonization (ICH) and Good Clinical Laboratory Practice (GCLP) outlined by the Research Quality Association (RQA), UK [29].

### Peptides

Peptides were synthesized at ~90% purity using high pressure liquid chromatography (HPLC) in the form of 15-mers with 11 overlapping amino acids. Nearly 8 peptide pools were used for the *in vitro* studies corresponding to Env (3 pools), Pol (3 pools) Nef-Tat (2 pool) and Gag (2 pool) (S2 Table). Cellular responses elicited by the HIV-peptides were evaluated at baseline and post MVA-boost in both groups. DMSO was used as mock-stimulus and 10 μg/ml of phytohemagglutinin (PHA) was used as the positive control.

### IFN-γ ELISPOT assay

HIV-specific cellular responses induced by the two candidate vaccines were initially evaluated by Interferon-Gamma Enzyme-Linked Immunospot (IFN-γ ELISpot) assay at baseline, one week after ADVAX-prime, at first MVA, last MVA, and 1 week after all MVA boosts in Group A. In Group B, responses were evaluated at baseline, at second MVA, last MVA, and 1 week after all MVA boosts. Cryopreserved PBMC obtained at the above set time points of vaccination were thawed, cells were washed twice with 10% Complete RPMI (Rosewell Park Memorial Institute) medium and rested overnight. Viability of the cells was evaluated using trypan blue dye exclusion method. Antihuman IFN-γ antibodys pre-coated ELISpot plates (kit 3410-2H, Mabtec Lab) were used for this assay. Briefly, 2x10^5^ PBMC were added to each well. 1.5 μg/ml of each Env, Gag, Pol, Nef-Tat peptide pools or Phytohemagglutinin at 10 μg/mL (PHA, Sigma-Aldrich), or equivalent volume of DMSO were added to the respective wells and plates were incubated in a 37°C humidified CO_2_ incubator for 16–24 hours. Cells were removed carefully and plates were washed 5 times with 200 μL of PBS/0.05% Tween 20 After washing, 100 μL of biotinylated anti-human IFN-γ antibody (Mabtec Lab 3420–6) was added to each wells and incubated at room temperature for 2–4 h. Plates were washed 5 times with 200 μL of PBS/0.05% Tween 20. Freshly prepared (100 μL/well) ABC complex (Vector Lab-PK6100) was added to each well and plates were incubated at room temperature for 1 hour. Plates were washed 5 times with 200 μL of PBS/0.05% Tween 20 (100 μL/well) AEC substrate (100 μL/well) (Vector Lab- SK4200) was added to each wells and plates were incubated at room temperature for 4 minutes. The reaction was stopped by gently washing under running tap water over a sink. Without delay, the plates were blotted dry over paper towels. Spots were counted using an automated AID ELISPOT reader (Autoimmun Diagnostika, Strassberg, Germany). The results were interpreted based on the following criteria. The total SFC/10^6^ PBMC had to satisfy the following conditions [30, 31] (i) The threshold value was fixed considering the distribution of baseline and placebo responses for the vaccinia-specific peptide pools. (ii) If the pools had more than one replicate, the coefficient of variation (standard deviation/mean) between the replicates had to be <70%. (iii) The average count had to be >4 times the average of background. (iv) The average of background had to be ≤55 SFC/10^6^ PBMC; assays with median of background counts >55 SFC/10^6^ PBMC were regarded as failures. If the baseline ELISPOT responses for any vaccinee were higher than 38 SFC/10^6^ PBMC, the responses corresponding to the peptide pools of that vaccinee were regarded as cross-reactive and hence were not considered for frequency calculations [26].

### Intracellular cytokine staining (ICS) and polychromatic flow cytometry (PFC)

The quantity and functionality of antigen-experienced T cells were assessed by flow-cytometry in terms of the nature and frequency of different cytokines they produced. PBMC collected at 2 weeks post first and last MVA, along with the corresponding baseline samples were analyzed for surface-antigen and cytokine-secretion adopting the standardized ICS assay in conjunction with polychromatic flow cytometry analysis as described previously [32]. (Production of three cytokines IFN-γ, TNF-α and IL-2 were examined. ICS assay was performed by stimulating PBMCs with a set of 554 HIV-1 peptides (S3 Table) (15-mers overlapping by 11 amino acids) grouped in to three pools: Env (202 peptides), Gag (119 peptides), and Pol (233 peptides). Staphylococcus Entero Toxin B was used as positive control and mock cultures (DMSO) was used as the negative control.)Briefly, cryopreserved PBMC were thawed and washed twice with 10% Complete RPMI and rested overnight. Viability of the cells was evaluated using trypan blue dye exclusion method. For each stimulation, 1 x 10^6^ PBMC were added to the corresponding tubes followed by costimulatory CD28/49d antibody at a concentration of 1 μg/mL. Env, Gag, Pol peptide (15-mers overlapping by 11 amino acids) pools at 1.5 μg/ml, 1 μg/mL staphylococcal enterotoxin B (SEB) (Sigma-Aldrich, India) as positive control or DMSO as mock. Brefeldin A (Sigma-Aldrich, India) was added at 5 μL/stimulation. Tubes were incubated in a 37°C humidified CO_2_ incubator for 6 hours. Stimulated cells were washed with 2 mL of Stain buffer (Becton-Dickinson). Aqua Amine Reactive Viability Dye (L34962) (Invitrogen, India) was added and incubated for 20 minutes at room temperature in the dark. After washing, anti-CD3 APCH7(SK7) (Becton-Dickinson), anti-CD4 BUV737 (RPA-T4) (Becton-Dickinson), anti-CD8 AF700 (RPA-T8) (Becton-Dickinson), anti-CD45RO BUV395 (UCHL1) (Becton-Dickinson), and anti-CCR7 PECY7(3D12) (Becton-Dickinson) were added, and stained intracellularly with IFN-γ APC B27 (Becton-Dickinson), IL2 PE MQ1-17HI2 (Becton-Dickinson) and TNF-α FITC (Mab11) along with BD Fixation/Permeabilization buffer (Becton-Dickinson) (S1 Table) for 20 minutes at room temperature. Cells were washed with perm/wash (Becton-Dickinson) and fixed with BD Fixation/Permeabilization buffer. A minimum of 500,000 events were acquired on a custom-built BD FACS Aria Sorp flow cytometer. The results were analyzed using FlowJo software v 10.5 (Tree star Inc., Ashland, Oregon, USA).

### Statistical analysis

Statistical analyses were performed using GraphPad Prism, version 5 (GraphPad Software, Inc., CA).Data were verified; normality was assessed and found to be non-normal distributed using Shapiro-Wilks test. Summary statistics are presented as percentage, median, and range. Mann–Whitney test was performed to compare vaccine induced cytokine responses against the peptide pools between the two groups. For all analyses, differences were considered significant if p value was <0.05.

## Results

### Induction of HIV-specific IFN-γ response

IFN-γ ELISpot assay was used to initially assess antigen-specific cellular responses at various time points. In Group A, ELISpot responses were assessed at the following time points: prevaccination, 2 weeks after first DNA vaccination, at second vaccination (1^st^ MVA), 1 week after second vaccination, at last vaccination, and 1 week after the last MVA boost. In Group B, ELISpot responses were assessed at the following time points: prevaccination, 2 weeks after first MVA vaccination, at second MVA vaccination, 1 week after second MVA vaccination, and 1 week after last MVA vaccination. The responses were tested against HIV-1 Env peptides (3 pools), Pol peptides (3 pools), Gag peptides (2 pools) and Nef-Tat peptides (2 pools).

A total of 96 frozen PBMC samples were tested using this method. The viability of the PBMC always exceeded 85–90%, and all samples were tested following overnight resting at 37°C post-thawing. All samples passed the PHA positive control criteria for quality control. IFN-γ ELISpot responses to up to three peptide pools were commonly observed in both the groups at multiple time points indicating that a good HIV-specific IFN-γ response was elicited early and persisted over time in the vaccine recipients.

The magnitude of the ELISpot response for the mock and PHA control for the 96 specimens were on an average 10 and 734 Spot Forming Cells (SFC)/million PBMC, with standard deviations (SD) of 9, and 195 SFC/million PBMC, respectively. IFN-γ ELISpot response to any of the peptide pools was detected in 5 of the 6 vaccinees (83%) in Group A; 6/6 vaccinees (100%) responded to Env, 4/6 vaccinees (66%) responded to Gag, 4/6 vaccinees (66%) responded to Pol and 2/6 vaccinees (33%) responded to Nef-Tat peptide pools after the MVA boost. In group B, IFN-γ ELISpot responses to any of the peptide pools was detected in 3/6 vacinees (50%); 3/6 vaccinees (50%) responded to Env, 2/6 vaccinees (35%) responded to Gag, 2/6 vaccinees (35%) responded to Pol and 1/6 vaccinees (16%) responded to Nef-Tat peptide pools.

At the time of last vaccination and 2 weeks post last vaccination, IFN-γ ELISpot responses to all three Env peptide pools (Env -1, Env 2 and Env -3), two Gag pool (Gag-1 and Gag-2) and three Pol pools (Pol-1, Pol-2 and Pol-3) were found to be significantly higher in Group A than in Group B (Last vaccination- Env -1: p = 0.055, Env -2:p = 0.010,Env -3:p = 0.004, Gag-1:p = 0.007,Gag-2:p = 0.054, Pol-1:p = 0.004, Pol-2:p = 0.055,Pol-3:p = 0.005 and 2 weeks post last vaccination- Env -1: p = 0.010, Env -2:p = 0.016, Env -3:p = 0.006,Gag-1:p = 0.010, Pol-1:p = 0.020, Pol-2:p = 0.007,Pol-3:p = 0.037). At last vaccination, 5/6 volunteers in Group A and 2/6 volunteers in Group B responded to the Env peptides; 4/6 volunteers in Group A and 2/6 in Group B responded to Gag peptide, and 4/6 (66%) volunteers in group A and 2/6 (33%) in Group B responded to the Pol peptides (Table 1).

**Table 1:** Median and range of positive IFN-γ ELISPOT responses (SFC/10^6^ PBMC) across different time points.

Table-1				
Peptide	Group A (n = 6)	Group B (n = 6)	Placebo (n = 4)	P Value *
pools	median (IQR)	median (IQR)	median (IQR)	
**Pre VAC**				
**ENV-1**	23.3 (15.0–25.0)	21.9 (16.7–23.3)	18.9 (11.7–29.7)	0.747
**ENV-2**	21.7 (20.0–33.3)	25.0 (16.7–25.6)	7.2 (5.6–11.7)	>0.950
**ENV-3**	18.3 (18.3–25.0)	17.8 (13.3–30.6)	22.5 (10.8–25.0)	>0.950
**GAG-1**	22.5 (20.0–25.0)	19.4 (15.0–23.3)	13.3 (6.7–21.9)	0.422
**GAG-2**	25.8 (16.7–33.3)	22.8 (16.7–26.1)	10.8 (7.5–15.0)	0.520
**N&T1**	20.8 (20.0–23.3)	16.7 (13.3–18.9)	11.7 (6.7–19.2)	0.092
**N&T2**	16.7 (10.0–21.7)	20.3 (16.7–23.3)	12.5 (7.5–24.2)	0.630
**POL-1**	21.7 (15.0–28.3)	22.8 (11.7–27.2)	19.7 (12.5–22.2)	0.936
**POL-2**	19.2 (16.7–23.3)	17.5 (13.3–23.3)	7.5 (3.3–17.5)	0.629
**POL-3**	25.0 (20.0–25.0)	27.2 (23.9–28.3)	13.3 (10.0–20.8)	0.196
**PHA**	589.2 (450.0–698.3)	362.5 (205.0–383.3)	861.7 (372.5–1310.0)	0.055
**Week II Post VAC I**				
**ENV-1**	194.2 (50.0–623.3)	17.2 (8.3–24.4)	20.1 (12.1–61.4)	0.025
**ENV-2**	52.5 (31.7–170.0)	28.9 (10.0–34.4)	12.8 (8.6–26.7)	0.173
**ENV-3**	35.8 (21.7–46.7)	23.1 (10.0–55.0)	20.3 (12.8–25.0)	0.423
**GAG-1**	111.7 (20.0–198.3)	38.3 (23.3–140.0)	23.3 (20.8–25.8)	0.749
**GAG-2**	40.0 (21.7–66.7)	30.0 (16.7–55.0)	30.8 (20.8–33.3)	0.630
**N&T1**	30.0 (11.7–33.3)	18.3 (16.7–78.3)	26.7 (17.5–32.5)	>0.950
**N&T2**	23.3 (21.7–28.3)	21.7 (11.7–41.7)	28.3 (26.7–32.5)	0.628
**POL-1**	65.0 (25.0–135.0)	28.9 (20.0–35.0)	13.6 (10.8–21.9)	0.262
**POL-2**	31.7 (21.7–55.0)	33.1 (21.7–160.0)	15.8 (11.1–20.8)	0.810
**POL-3**	32.5 (25.0–86.7)	23.1 (18.3–31.7)	22.5 (15.3–31.7)	0.423
**PHA**	498.3 (430.0–765.0)	557.5 (258.3–1060.0)	680.8 (247.5–1091.7)	0.873
**VAC II**				
**ENV-1**	199.2 (100.0–265.0)	24.4 (15.0–40.0)	25.6 (18.1–48.3)	0.030
**ENV-2**	130.0 (75.0–295.0)	28.3 (18.3–35.0)	37.5 (14.2–63.3)	0.078
**ENV-3**	168.3 (35.0–286.7)	21.7 (18.3–23.9)	48.3 (38.3–57.5)	0.149
**GAG-1**	278.3 (195.0–420.0)	76.7 (30.0–280.0)	32.5 (16.7–55.0)	0.150
**GAG-2**	186.7 (126.7–376.7)	57.5 (31.7–76.7)	19.2 (15.0–28.3)	0.005
**N&T1**	68.3 (33.3–171.7)	26.7 (20.0–58.3)	30.8 (22.5–47.5)	0.109
**N&T2**	75.0 (25.0–195.0)	41.2 (21.7–63.3)	27.5 (7.5–76.7)	0.378
**POL-1**	52.5 (26.7–116.7)	37.8 (15.6–79.4)	26.7 (21.7–32.5)	0.522
**POL-2**	122.5 (41.7–143.3)	44.4 (26.1–110.0)	35.8 (30.0–53.3)	0.423
**POL-3**	208.3 (21.7–306.7)	30.8 (18.3–46.7)	39.2 (25.8–46.7)	0.109
**PHA**	543.3 (440.0–728.3)	635.0 (633.3–655.0)	460.0 (402.5–556.7)	0.423
**Week I Post VAC II**				
**ENV-1**	392.5 (143.3–613.3)	217.5 (32.8–623.3)	44.2 (16.9–120.0)	0.522
**ENV-2**	225.0 (51.7–585.0)	188.3 (31.1–361.7)	90.0 (22.8–146.7)	0.337
**ENV-3**	212.5 (115.0–451.7)	38.9 (30.0–46.7)	45.8 (25.3–63.3)	0.109
**GAG-1**	159.2 (41.7–266.7)	142.5 (40.0–218.3)	26.7 (20.8–45.0)	0.873
**GAG-2**	189.2 (81.7–316.7)	308.3 (186.7–455.0)	102.5 (52.5–140.0)	0.262
**N&T1**	135.0 (113.3–211.7)	92.5 (33.3–120.0)	61.7 (30.0–118.3)	0.337
**N&T2**	50.0 (33.3–81.7)	44.3 (21.7–73.3)	36.7 (18.3–50.0)	0.522
**POL-1**	144.2 (66.7–145.0)	49.2 (24.4–85.0)	36.7 (20.3–58.3)	0.149
**POL-2**	138.3 (65.0–213.3)	32.5 (21.7–126.7)	44.2 (22.8–55.8)	0.173
**POL-3**	69.2 (56.7–165.0)	40.6 (16.7–218.3)	21.7 (11.1–36.7)	0.749
**PHA**	440.0 (296.7–511.7)	632.5 (523.3–780.0)	521.7 (406.9–595.0)	0.045
**Last Vaccination**				
**ENV-1**	73.3 (61.7–101.7)	40.3 (13.3–63.3)	44.2 (16.4–83.3)	0.055
**ENV-2**	100.8 (33.3–213.3)	22.5 (13.3–26.7)	48.3 (34.7–81.7)	0.010
**ENV-3**	155.0 (88.3–246.7)	23.9 (15.0–30.0)	43.3 (38.3–60.0)	0.004
**GAG-1**	170.8 (70.0–410.0)	18.3 (13.3–35.0)	40.8 (12.5–73.3)	0.007
**GAG-2**	144.2 (41.7–295.0)	21.7 (5.0–43.3)	16.7 (8.3–44.2)	0.054
**N&T1**	52.5 (30.0–118.3)	26.7 (21.7–41.7)	38.3 (18.3–58.3)	0.229
**N&T2**	30.8 (15.0–50.0)	18.3 (5.0–61.7)	38.3 (28.3–75.0)	0.522
**POL-1**	190.8 (60.0–223.3)	35.3 (17.2–39.4)	35.6 (18.1–45.8)	0.004
**POL-2**	97.5 (41.7–220.0)	27.2 (16.7–36.7)	37.5 (14.2–90.0)	0.055
**POL-3**	67.5 (45.0–95.0)	14.2 (10.0–20.6)	26.7 (13.3–35.8)	0.005
**PHA**	406.7 (391.7–566.7)	950.0 (475.0–1813.3)	716.7 (408.3–888.3)	0.109
**Week I Post Last Vaccination**				
**ENV-1**	457.5 (125.0–473.3)	29.2 (22.8–73.3)	94.2 (18.1–196.7)	0.010
**ENV-2**	210.0 (73.3–486.7)	37.5 (26.1–66.7)	51.1 (11.9–123.3)	0.016
**ENV-3**	123.3 (66.7–246.7)	27.2 (21.1–30.0)	22.8 (10.3–64.2)	0.006
**GAG-1**	258.3 (85.0–335.0)	32.5 (30.0–45.0)	20.8 (13.3–41.7)	0.010
**GAG-2**	118.3 (66.7–278.3)	54.2 (25.0–75.0)	14.2 (6.7–34.2)	0.200
**N&T1**	55.8 (23.3–113.3)	26.7 (23.3–55.0)	16.7 (6.7–27.5)	0.333
**N&T2**	49.2 (23.3–101.7)	35.8 (20.0–41.7)	25.8 (22.5–33.3)	0.421
**POL-1**	114.2 (55.0–138.3)	26.7 (21.1–46.7)	48.6 (13.6–125.8)	0.020
**POL-2**	74.2 (46.7–116.7)	21.9 (20.0–23.3)	16.9 (15.3–63.3)	0.007
**POL-3**	102.5 (71.7–135.0)	18.6 (13.3–36.1)	24.7 (15.8–58.1)	0.037
**PHA**	397.5 (221.7–683.3)	680.0 (578.3–903.3)	474.2 (339.2–525.0)	0.150

(* Mann-Whitney Test was used to compare group A and group B at 5% level of significance)

The IFN-γ response in Group A was significantly higher than in Group B at all time points, suggesting that DNA vaccination primed the immune system for a long-term cell mediated immune response.

### Intracellular cytokine production

Poly Chromatic Flowcytometry was employed to characterize the T cell phenotypes and polyfunctionality prior to vaccination as well as at two weeks post second and final booster in Groups A and B. The gating strategy employed is shown in S1 Fig.

**Fig 2:**
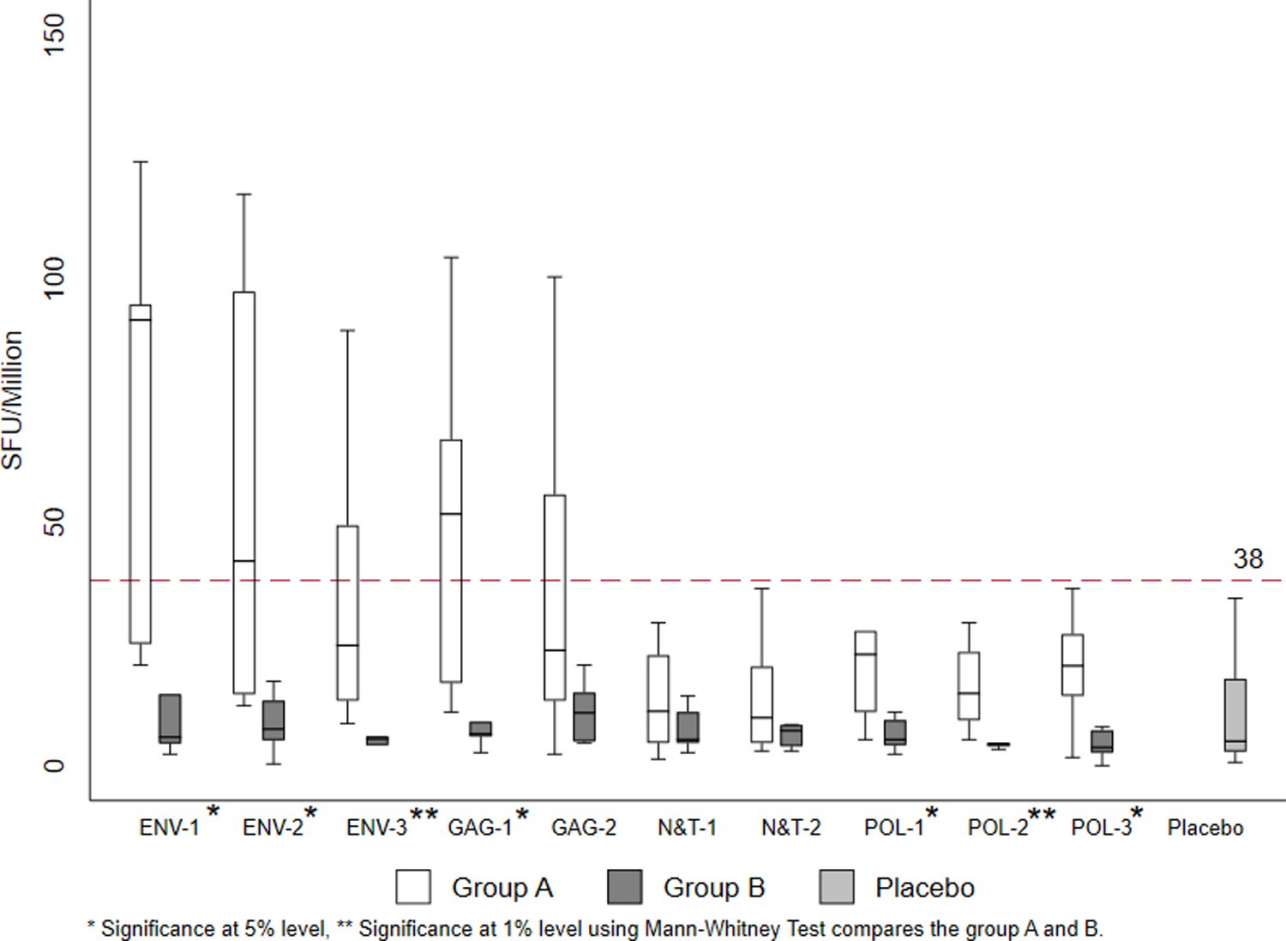
Frequency of positive IFN-γ ELISPOT responses. Magnitude of IFN-γ ELISPOT response (SFC/106 PBMC) and responder rate (%)at one week post last vaccination to Env, Gag, Pol and Nef /Tat peptides (group A: after 2 doses of DNA and 2 dose of MVA; group B after 3 doses of MVA; placebo results are from groups A and B combined).

In Group A, the ELISpot response to Env peptides was significantly higher than for Gag and Pol. However, in Group B, the magnitude of the response was overall lower and there was no apparent hierarchy in response to Env, Gag and Pol peptides (Fig 2). The response to Nef-Tat peptides was very minimal in both the groups. None of the volunteers who received placebo responded to any of the HIV-1 peptides.

**Fig 3:**
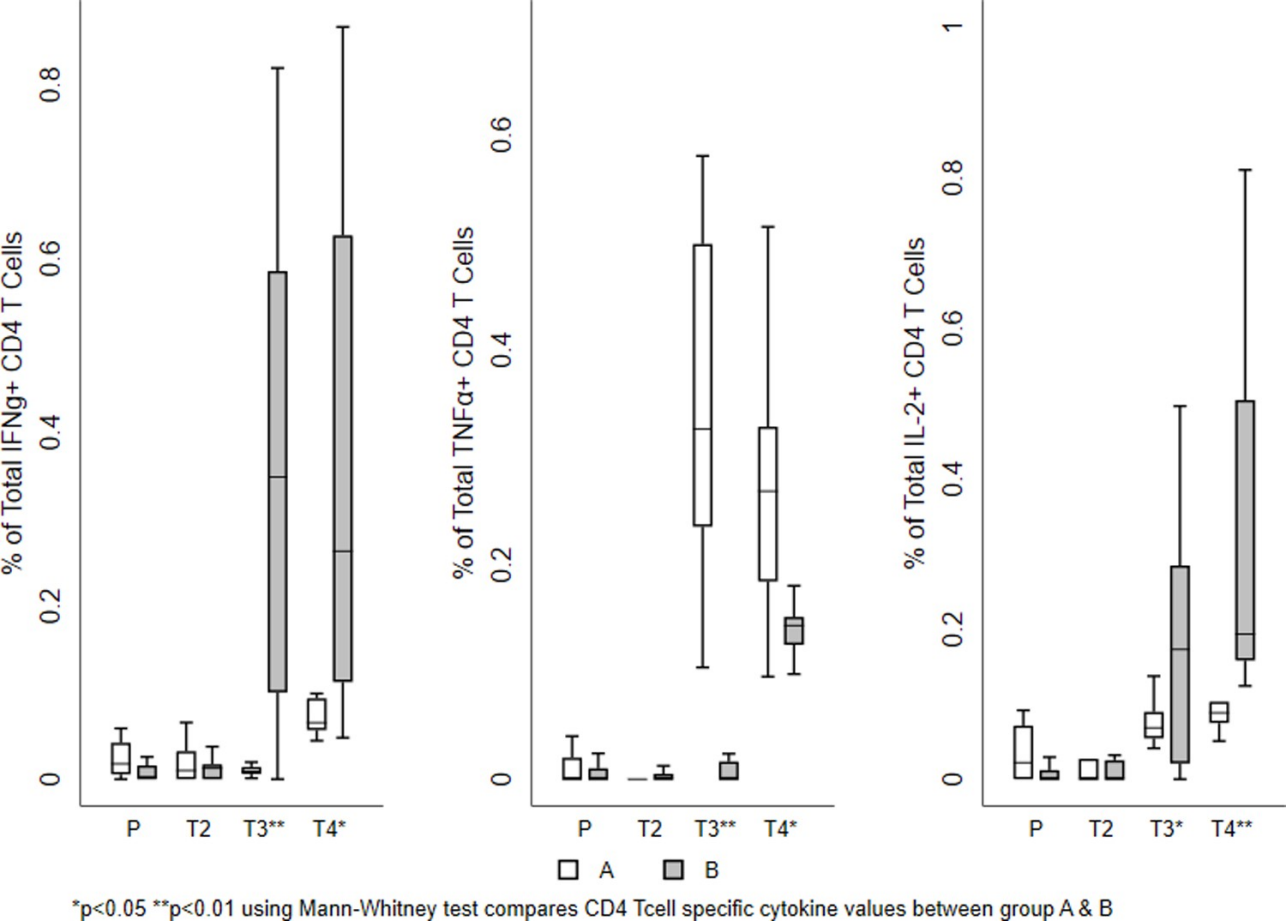
Vaccine-induced HIV-1-specific CD4^+^ T cell responses. Frequency of vaccine-specific CD4+ T cells measured at different time points. The mean value of the total responses (Env, Gag, and Pol) obtained for each T cell sub population is shown. (A) Frequency of IFN-γ positive vaccine induced T cell subsets. (B) Frequency of TNF-α producing vaccine induced T cell subsets. (C) Frequencies of IL-2 producing vaccine induced T cells sub populations. The box plots show the distribution of responses in positive responders only. The boxes indicate the median (solid line), mean (dashed line), and interquartile range (IQR). P values for significant differences were determined using the Mann-Whitney T test and are represented using the symbol * (Note: P-Placebo, T2-Prevaccination, T3-Second week after first MVA vaccination, T4-Second week after last MVA vaccination).

**Fig 4:**
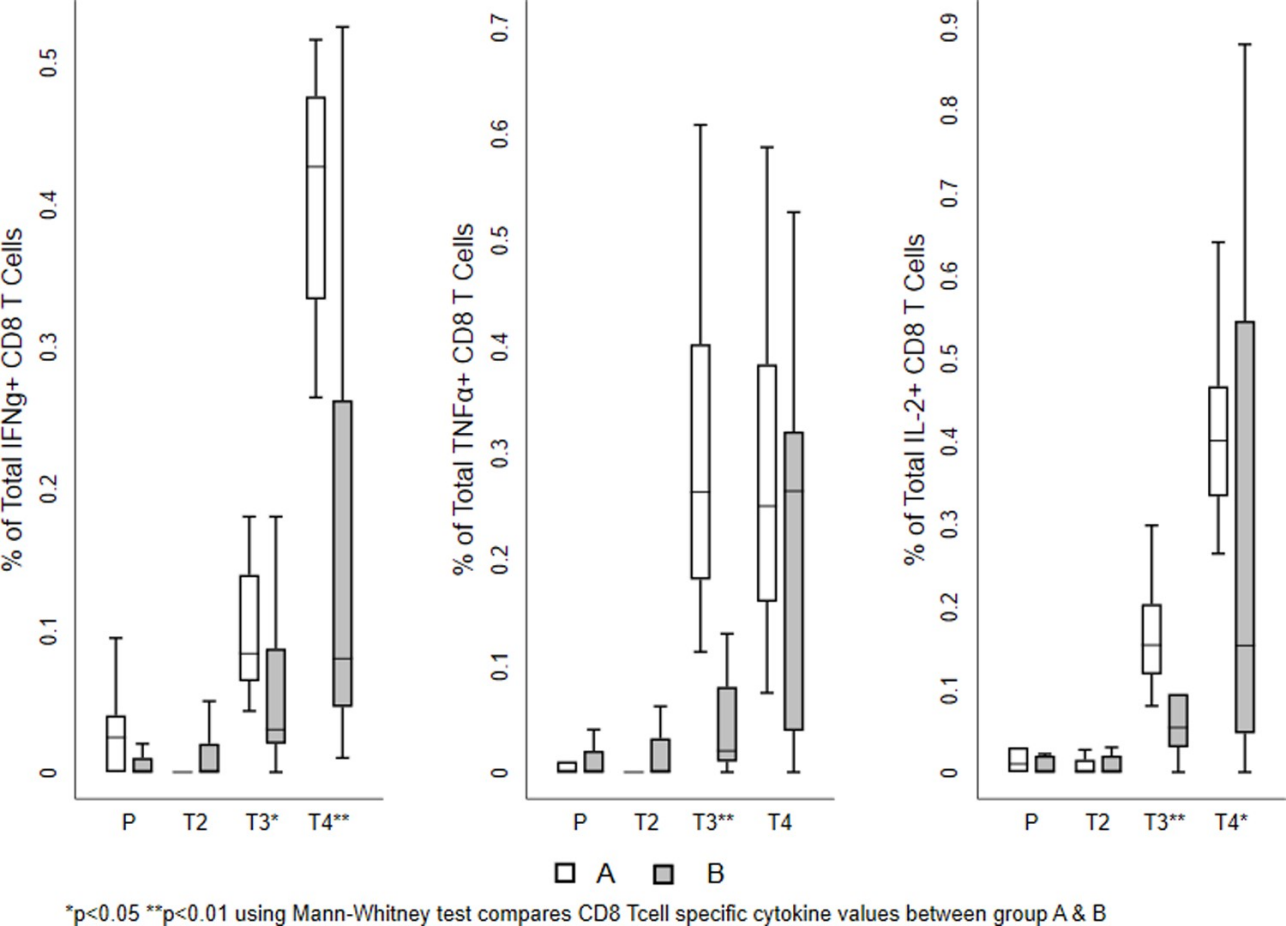
Vaccine-induced HIV-1-specific CD8^+^ T cell responses. Frequency of vaccine-specific CD8^+^ T cells at different time points. The mean value of the total responses (Env, Gag, and Pol) obtained for each T cell sub population is shown. (A) Frequency of IFN-γ positive vaccine induced T cell subsets. (B) Frequency of TNF-α positive vaccine induced T cell subsets. (C) Frequency of IL-2 positive vaccine induced T cell sub populations. The box plots show the distribution of responses in positive responders only. The boxes indicate the median (solid line), mean (dashed line), and interquartile range (IQR). P values for significant differences were determined using the Mann-Whitney T test and are represented using the symbol *. (Note: P-Placebo, T2-Prevaccination, T3-Second week after first MVA vaccination, T4-Second week after last MVA vaccination).

The mean values of the total responses obtained for each T cell subset shown in Figs 3 and 4. The magnitude of the total vaccine specific response was similar in both the groups at the two time points investigated. Among the cytokine-producing CD8^+^ T cells, IFN-γ and IL-2 production was more predominant than TNF-α in both the groups. On the other hand, TNF-α and IL-2 production was higher in the CD4^+^ T cells. Cumulative analysis of the data demonstrated that vaccine induced T cell responses were balanced and significantly higher in the vaccine recipients as compared to the placebo group (Figs 3 and 4 and Table 2). Most of the T cells were of the central memory or effector phenotype (S1 Fig).

**Table 2:** Vaccine responsiveness based on cytokine positive ICS assay.

**IFN-γ**							
**VACCINATION GROUP**	**ANTIGEN**	**AT PRE VACCINATION**		**AT 2 Wks. POST FIRST MVA**		**AT 2 Wks. POST LAST MVA**	
		**CD4**^**+**^**T cells**	**CD8**^**+**^**T cells**	**CD4**^**+**^**T cells**	**CD8**^**+**^**T cells**	**CD4**^**+**^**T cells**	**CD8**^**+**^**T cells**
**GROUP A**	ENV	1/6 (17%)	0/6 (0%)	3/6 (50%)	6/6 (100%)	6/6 (100%)	6/6 (100%)
	GAG	3/6 (50%)	1/6 (17%)	1/6 (17%)	6/6 (100%)	6/6 (100%)	6/6 (100%)
	POL	3/6 (50%)	1/6 (17%)	0/6 (0%)	6/6 (100%)	6/6 (100%)	6/6 (100%)
**GROUP B**	ENV	4/6 (67%)	1/6 (17%)	6/6 (100%)	6/6 (100%)	6/6 (100%)	6/6 (100%)
	GAG	4/6 (67%)	3/6 (50%)	6/6 (100%)	5/6 (83%)	6/6 (100%)	6/6 (100%)
	POL	1/6 (17%)	1/6 (17%)	2/6 (33%)	3/6 (50%)	6/6 (100%)	5/6 (83%)
**PLACEBO**	ENV	0/3 (0%)	0/3 (0%)	0/3 (0%)	0/3 (0%)	0/3 (0%)	0/3 (0%)
	GAG	0/3 (0%)	0/3 (0%)	0/3 (0%)	0/3 (0%)	0/3 (0%)	0/3 (0%)
	POL	0/3 (0%)	0/3 (0%)	0/3 (0%)	0/3 (0%)	0/3 (0%)	0/3 (0%)
**IL2**							
**VACCINATION GROUP**	**ANTIGEN**	**AT PRE VACCINATION**		**AT 2 Wks. POST FIRST MVA**		**AT 2 Wks. POST LAST MVA**	
		**CD4**^**+**^**T cells**	**CD8**^**+**^**T cells**	**CD4**^**+**^**T cells**	**CD8**^**+**^**T cells**	**CD4**^**+**^**T cells**	**CD8**^**+**^**T cells**
**GROUP A**	ENV	1/6 (17%)	1/6 (17%)	6/6 (100%)	6/6 (100%)	6/6 (100%)	6/6 (100%)
	GAG	1/6 (17%)	2/6 (33%)	6/6 (100%)	6/6 (100%)	6/6 (100%)	6/6 (100%)
	POL	3/6 (50%)	1/6 (17%)	6/6 (100%)	6/6 (100%)	6/6 (100%)	6/6 (100%)
**GROUP B**	ENV	3/6 (50%)	1/6 (17%)	5/6 (83%)	6/6 (100%)	6/6 (100%)	5/6 (83%)
	GAG	1/6 (17%)	3/6 (50%)	6/6 (100%)	6/6 (100%)	6/6 (100%)	6/6 (100%)
	POL	3/6 (50%)	2/6 (33%)	5/6 (83%)	3/6 (50%)	6/6 (100%)	5/6 (83%)
**PLACEBO**	ENV	0/3 (0%)	0/3 (0%)	0/3 (0%)	0/3 (0%)	0/3 (0%)	0/3 (0%)
	GAG	0/3 (0%)	0/3 (0%)	0/3 (0%)	0/3 (0%)	0/3 (0%)	0/3 (0%)
	POL	0/3 (0%)	0/3 (0%)	0/3 (0%)	0/3 (0%)	0/3 (0%)	0/3 (0%)
**TNF-α**							
**VACCINATION GROUP**	**ANTIGEN**	**AT PRE VACCINATION**		**AT 2 Wks. POST FIRST MVA**		**AT 2 Wks. POST LAST MVA**	
		**CD4**^**+**^**T cells**	**CD8**^**+**^**T cells**	**CD4**^**+**^**T cells**	**CD8**^**+**^**T cells**	**CD4**^**+**^**T cells**	**CD8**^**+**^**T cells**
**GROUP A**	ENV	1/6 (17%)	1/6 (17%)	6/6 (100%)	6/6 (100%)	6/6 (100%)	6/6 (100%)
	GAG	0/6 (0%)	0/6 (0%)	6/6 (100%)	6/6 (100%)	6/6 (100%)	6/6 (100%)
	POL	1/6 (17%)	2/6 (33%)	6/6 100%)	6/6 (100%)	6/6 (100%)	6/6 (100%)
**GROUP B**	ENV	2/6 (33%)	3/6 (50%)	3/6 (50%)	3/6 (50%)	6/6 (100%)	6/6 (100%)
	GAG	0/6 (0%)	2/6 (33%)	2/6 (33%)	5/6 (83%)	6/6 (100%)	6/6 (100%)
	POL	1/6 (17%)	2/6 (33%)	0/6 (0%)	5/6 (83%)	6/6 (100%)	5/6 (83%)
**PLACEBO**	ENV	0/3 (0%)	0/3 (0%)	0/3 (0%)	0/3 (0%)	0/3 (0%)	0/3 (0%)
	GAG	0/3 (0%)	0/3 (0%)	0/3 (0%)	0/3 (0%)	0/3 (0%)	0/3 (0%)
	POL	0/3 (0%)	0/3 (0%)	0/3 (0%)	0/3 (0%)	0/3 (0%)	0/3 (0%)

The frequency of both CD4^+^ and CD8^+^ T cells producing the indicated cytokine increased post-vaccination; the responses were predominantly directed to Env and Gag peptides in both the groups. Vaccine-specific CD8^+^ T cell responses were much higher than CD4^+^ T cell responses in terms of multi-functionality. MVA booster response was significantly higher in CD8^+^ T cells in Group A than in Group B (Table 2).

**Fig 5:**
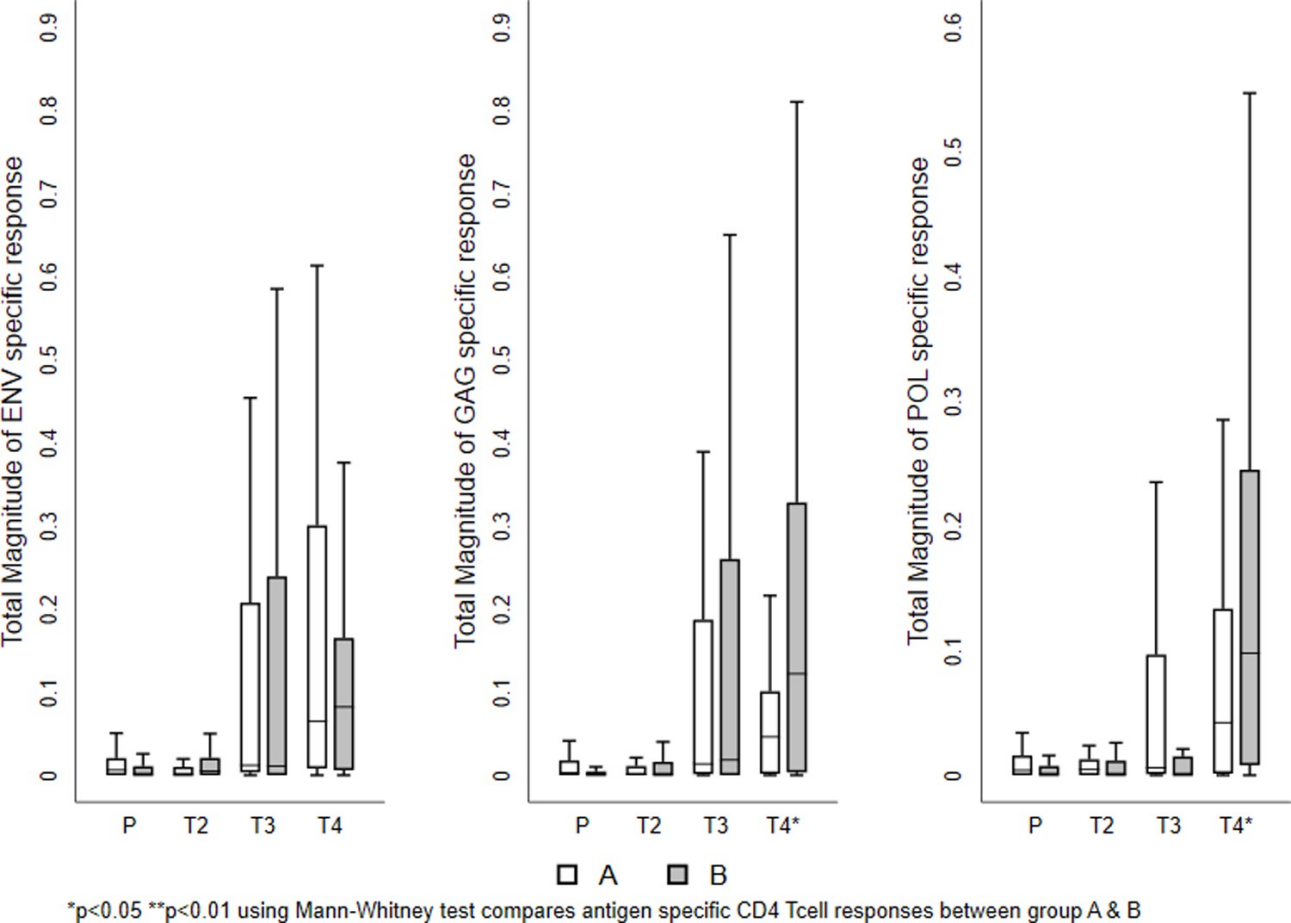
Vaccine-induced antigen-specific CD4^+^ T cell response. The mean values of the total responses (Env, Gag and Pol) are shown. A) Frequency of Env-specific CD4^+^ T cells (B) Frequency of Gag-specific CD4^+^ T cells. (C) Frequency of Pol-specific CD4^+^ T cells. The box plots show the distribution of responses in positive responders only. The boxes indicate the median (solid line), mean (dashed line), and interquartile range (IQR). P values were determined using Mann-Whitney T test. (Note: P-Placebo, T2-Prevaccination, T3-Second week after first MVA vaccination, T4-Second week after last MVA vaccination).

**Fig 6:**
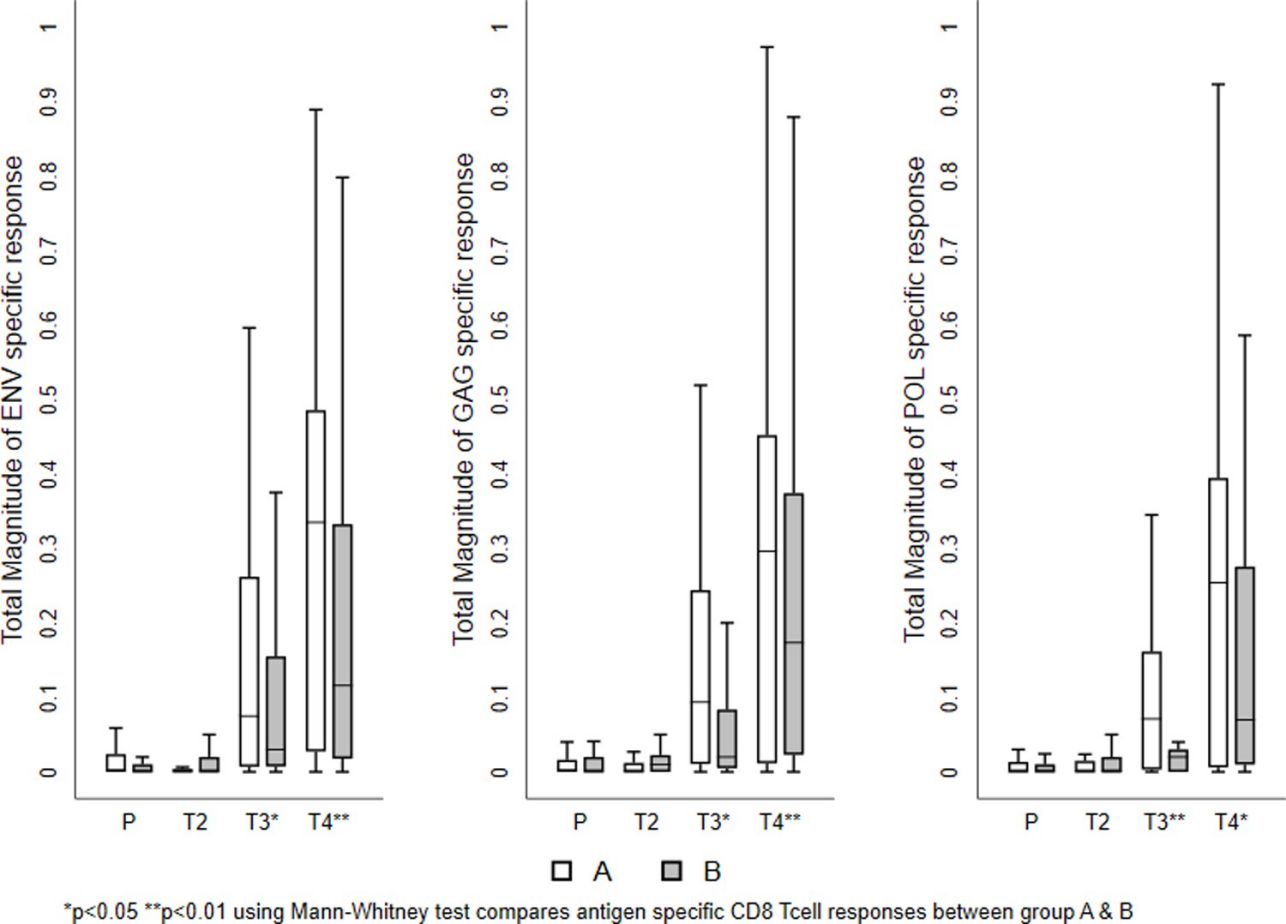
Vaccine-induced antigen -specific CD8^+^ T cell response. The mean values of the total responses (Env, Gag and Pol) are shown. A) Frequency of Env-specific CD8^+^ T cells (B) Frequency of Gag-specific CD8^+^ T cells. (C) Frequency of Pol-specific CD8^+^ T cells. The box plots show the distribution of responses in positive responders only. The boxes indicate the median (solid line), mean (dashed line), and interquartile range (IQR). P values were determined using Mann-Whitney T test. (Note: P-Placebo, T2-Prevaccination, T3-Second week after first MVA vaccination, T4-Second week after last MVA vaccination).

All samples that had a higher-magnitude of response at 2 weeks post vaccination were found to belong to Group A. The responses were predominantly seen in CD8^+^ T cells cytokine to Env and Gag peptides. Similarly, bi-functional IFN-γ+ TNF-α+ or IFN-γ+ IL2+ or IL2+TNF-α+ and mono functional IFN-γ+ or TNF-α+ or IL-2+ cells, were significantly higher in vaccinees who received the heterologous regimen than in those who received the homologous regimen. However, the frequency of polyfunctional CD8+ T cells expressing IFN-γ+ TNF-α+ and IL-2+ were significantly higher in Group B than in those received homologous vaccination (Figs 5 and 6 and Tables 3 and 4 and S4); however, the frequency of poly functional T cells were found to be very small.

**Table 3:** Vaccine-induced peptide specific CD8^+^ T cell cytokine responses across the period of vaccination.

**Cytokine**	**Antigen and Group**			
	**ENV**			P value *
	**Placebo (n = 4)** **median (IQR)**	**Group A (n = 6)** **median (IQR)**	**Group B (n = 6)** **median (IQR)**	
IFN-γ^+^ TNF-α^+^ IL-2^+^ (Poly functional)	0.0046	0.0083	0.0180	0.2105
	(0–0.04)	(0–0.03699)	(0–0.16)	
IFN-γ^+^ TNF-α^+^ (Bi-functional)	0.0000	0.0000	0.0085	0.0100
	(0–0.0277)	(0–0.32)	(0–0.07)	
IFN-γ^+^ IL-2^+^ (Bi-functional)	0.0100	0.0705	0.0300	0.7380
	(0–0.0427)	(0–0.648)	(0–0.481)	
IFN-γ^+^ (Mono functional)	0.0050	0.0762	0.1000	0.7857
	(0–0.0945)	(0–0.834)	(0–0.743)	
IL-2^+^ (Mono functional)	0.0050	0.1375	0.0524	0.0795
	(0–0.09)	(0–0.758)	(0–0.37)	
IL-2^+^ TNF-α^+^ (Bi-functional)	0.0050	0.0063	0.0026	0.3816
	(0–0.0468)	(0–0.0787)	(0–0.3)	
TNF-α^+^ (Mono functional)	0.0000	0.2190	0.0302	0.0518
	(0–0.02)	(0–0.587)	(0–0.361)	
**Cytokine**	**GAG**			P value*
IFN-γ^+^ TNF-α^+^ IL-2^+^ (Poly functional)	0.0043	0.0061	0.0152	0.0280
	(0–0.041)	(0–0.0366)	(0–0.195)	
IFN-γ^+^ TNF-α^+^ (Bi-functional)	0.0007	0.0000	0.0117	0.0777
	(0–0.0189)	(0–0.0201)	(0–0.211)	
IFN-γ^+^ IL-2^+^ (Bi-functional)	0.0105	0.0882	0.0200	0.0930
	(0–0.05)	(0.00438–0.57)	(0–0.534)	
IFN-γ^+^ (Mono functional)	0.0100	0.0979	0.0380	0.0897
	(0–0.2)	(0–0.698)	(0–0.31)	
IL-2^+^ (Mono functional)	0.0000	0.1905	0.0842	0.9747
	(0–0.344)	(0–0.64)	(0–0.879)	
IL-2^+^ TNF-α^+^ (Bi-functional)	0.0018	0.0108	0.0000	0.0097
	(0–0.0122)	(0–0.0511)	(0–0.0309)	
TNF-α^+^ (Mono functional)	0.0000	0.2440	0.0709	0.8478
	(0–0.4)	(0–0.608)	(0–0.96)	
**Cytokine**	**POL**			P value*
IFN-γ^+^ TNF-α^+^ IL-2^+^ (Poly functional)	0.0010	0.0031	0.0200	0.0284
	(0–0.059)	(0–0.0225)	(0–0.248)	
IFN-γ^+^ TNF-α^+^ (Bi-functional)	0.0000	0.0000	0.0088	0.0695
	(0–0.0169)	(0–0.0223)	(0–0.03)	
IFN-γ^+^ IL-2^+^ (Bi-functional)	0.0000	0.0686	0.0200	0.0708
	(0–0.0434)	(0.00713–0.366)	(0–0.586)	
IFN-γ^+^ (Mono functional)	0.0050	0.0896	0.0150	0.0149
	(0–0.0719)	(0–0.429)	(0–0.101)	
IL-2^+^ (Mono functional)	0.0000	0.1420	0.0200	0.0262
	(0–0.135)	(0–0.466)	(0–0.55)	
IL-2^+^ TNF-α^+^ (Bi-functional)	0.0000	0.0058	0.0000	0.0112
	(0–0.0217)	(0–0.0448)	(0–0.04)	
TNF-α^+^ (Mono functional)	0.0000	0.1555	0.0280	0.1593
	(0–0.05)	(0–0.51)	(0–0.526)	

(*Mann-Whitney Test was used to compare group A and group B at 5% level of significance)

**Table 4:** Vaccine-induced peptide specific CD4^+^ T cell cytokine responses across the period of vaccination.

**Cytokine**	**Antigen and Group**			
	**ENV**			P value*
	**Placebo (n = 4)** **median (IQR)**	**Group A (n = 6)** **median (IQR)**	**Group B (n = 6)** **median (IQR)**	
IFN-γ^+^ TNF-α^+^ IL-2^+^ (Poly functional)	0.0004	0.0016	0.0000	0.6601
	(0–0.021)	(0–0.03)	(0–0.149)	
IFN-γ^+^ TNF-α^+^ (Bi-functional)	0.0000	0.0004	0.0000	0.8640
	(0–0.011)	(0–0.09)	(0–0.02)	
IFN-γ^+^ IL-2^+^ (Bi-functional)	0.0153	0.0110	0.0266	0.3334
	(0–0.0483)	(0–0.79)	(0–0.24)	
IFN-γ^+^ (Mono functional)	0.0156	0.0224	0.1007	0.0686
	(0–0.0642)	(0–0.926)	(0–0.833)	
IL-2^+^ (Mono functional)	0.0028	0.0696	0.1570	0.1703
	(0–0.0916)	(0–0.84)	(0–0.314)	
IL-2^+^ TNF-α^+^ (Bi-functional)	0.0026	0.0073	0.0000	0.0021
	(0–0.0314)	(0–0.0159)	(0–0.04)	
TNF-α^+^ (Mono functional)	0.0000	0.2150	0.0184	0.0246
	(0–0.04)	(0–0.613)	(0–0.55)	
**Cytokine**	**GAG**			P value*
IFN-γ^+^ TNF-α^+^ IL-2^+^ (Poly functional)	0.0000	0.0000	0.0000	0.7774
	(0–0.0127)	(0–0.03)	(0–0.252)	
IFN-γ^+^ TNF-α^+^ (Bi-functional)	0.0000	0.0011	0.0000	0.2051
	(0–0.0038)	(0–0.0175)	(0–0.02)	
IFN-γ^+^ IL-2^+^ (Bi-functional)	0.0029	0.0113	0.0234	0.3261
	(0–0.0245)	(0–0.0473)	(0–0.245)	
IFN-γ^+^ (Mono functional)	0.0053	0.0237	0.2625	0.0032
	(0–0.0585)	(0–0.0986)	(0–0.866)	
IL-2^+^ (Mono functional)	0.0000	0.0783	0.2070	0.0222
	(0–0.404)	(0–0.137)	(0–0.81)	
IL-2^+^ TNF-α^+^ (Bi-functional)	0.0030	0.0140	0.0000	<0.001
	(0–0.0414)	(0–0.0351)	(0–0.0259)	
TNF-α^+^ (Mono functional)	0.0000	0.2525	0.0095	0.0225
	(0–0.313)	(0–0.58)	(0–0.252)	
**Cytokine**	**POL**			P value*
IFN-γ^+^ TNF-α^+^ IL-2^+^ (Poly functional)	0.0000	0.0005	0.0000	0.7609
	(0–0.01)	(0–0.0164)	(0–0.0978)	
IFN-γ^+^ TNF-α^+^ (Bi-functional)	0.0006	0.0005	0.0000	0.9454
	(0–0.012)	(0–0.00902)	(0–0.0269)	
IFN-γ^+^ IL-2^+^ (Bi-functional)	0.0075	0.0081	0.0272	0.3646
	(0–0.0266)	(0–0.04)	(0–0.115)	
IFN-γ^+^ (Mono functional)	0.0110	0.0210	0.0129	0.8978
	(0–0.05)	(0–0.723)	(0–0.64)	
IL-2^+^ (Mono functional)	0.0000	0.0729	0.0735	0.7157
	(0–0.0727)	(0–0.648)	(0–0.734)	
IL-2^+^ TNF-α^+^ (Bi-functional)	0.0008	0.0047	0.0000	<0.001
	(0–0.016)	(0–0.0237)	(0–0.01)	
TNF-α^+^ (Mono functional)	0.0025	0.1525	0.0000	0.0114
	(0–0.02)	(0–0.618)	(0–0.206)	

(*Mann-Whitney Test was used to compare group A and group B at 5% level of significance)

The mean values of the total response to Env, Gag, and Pol peptides in each T cell population are shown in Figs 5 and 6. The CD4^+^ T cell response was essentially directed against the Env and Gag peptide pools, whereas the CD8^+^ T cell response was evenly distributed between the Env, Gag and Pol peptides (Tables 3 and 4). Significantly higher CD8^+^ T cells responses were seen in vaccinees at all-time points (S4 Table).

## Discussion

Understanding the induction of long-lived HIV-specific immune responses is critical for evaluating the efficacy of a vaccine candidate. Memory T cell response is associated with protection and is therefore a critical component of vaccines that induce protective immune responses [33]. Different patterns of immune response mediated by CD4+ and CD8+ T cells are induced by different vaccine modalities. However, the ability of a vaccine to elicit both humoral and cell-mediated immune responses contributes greatly to the efficacy of a given vaccine. Recent research in HIV vaccines has focused largely on the induction of T cell immunity due to failure of vaccines to induce neutralizing antibodies [34].

Cell-mediated immune responses play a vital role in the clearance of viral infections. CD8^+^ T cells control viral infection by direct lysis of infected cells and/or through production of antiviral cytokines. CD4^+^ T cells also help in controlling viral infection through the secretion of cytokines that promote and maintain a strong antiviral CD8^+^ T cell response, and help in the generation of an effective B cell response. An inverse correlation has been observed between frequency of polyfunctional antigen-specific CD8^+^ T cells and viral load in individuals with chronic viral infections [35–37]. The recognition of viral epitopes by CD8^+^ T cells is of great importance, and much effort has gone into the identification and design of specific peptide epitopes that can induce a potent antiviral T cell response [38].

DNA vaccines in combination with other vector-based vaccines are thought to hold the future prospects of vaccination [39]. DNA vaccines are known to act better as prime rather than boost when combined with other modalities, and the nature of the immune response generated by DNA vaccination is determined by the nature of the boost [40]. A CD4^+^ T cell biased response that provides T cell help for an antibody response is observed when proteins are used as the boost, while a predominant CD8^+^ T cell response is generated when viral vectors are employed for boosting [41]. HIV DNA vaccines are usually poor immunogens when used alone in humans but are capable of efficiently priming immune responses when used in prime boost regimens with live recombinant HIV vaccine [37]. Many of the recent HIV vaccine trials have employed the heterologous prime boost approach, that usually employs HIV DNA plasmids or recombinant Env glycoproteins in combination with a non-replicating viral vector based vaccine. HIV vaccines based on plasmid DNA and/or live recombinant virus vectors mainly stimulate cellular immune responses. T cell vaccines are usually not expected to prevent acquisition of infection, but prevent development of disease by reducing the viral load [42]. New approaches that elicit protective immunity by manipulating the T cell repertoire may be particularly useful for designing a successful therapeutic HIV vaccine.

Interestingly, the preliminary studies on the P001 Phase I HIV-1 vaccine trial that tested the safety and immunogenicity of heterologous prime-boost immunization regimen with DNA prime (ADVAX) and MVA (TBC-M4) boost, and a homologous prime and boost regimen with MVA alone, revealed that vaccination resulted in enhancement of antibody and IFN-γ responses [27]. The study further reported that although the response appeared to be significantly higher in the DNA prime/MVA boost group following first MVA boost, the effect lasted only for a short time, implying that both the DNA/MVA heterologous prime-boost and the homologous MVA regimens were immunologically comparable [27]. The present study describes a detailed evaluation of the vaccine-induced cell mediated immune responses in archived PBMC using multicolor flow cytometry.

We first evaluated vaccine-induced IFN-γ production using the ELISpot assay and found that vaccination induced significantly higher IFN-γ responses, particularly in response to Env and Gag antigens, clearly indicating that vaccination resulted in successful priming of the immune system. Subsequently we published another report describing in detail the humoral immune responses induced by this vaccine [43]. The magnitude of the IFN-γ response was found to be higher in volunteers who received the heterologous regimen, as compared to those who received the homologous regimen. After the last vaccination, the magnitude of the IFN-γ response to Env was observed to be greater than for Gag or Pol in Group A. In Group B the magnitude of the response was lower and there was no apparent hierarchy of responses to Env, Gag or Pol. There was a very limited response to the Nef-Tat pool in both groups and the response was sporadic. Similar findings were reported from the UK trial that tested the same vaccine constructs [26].

We then investigated mono functional and multi-functional T cell responses using polychromatic flowcytometry, since CD8^+^ T cell responses with a robust multi-functional component have been strongly linked to better disease outcome. Further we examined, the relationship between the T central memory (T_CM_) response and polyfunctionality, since polyfunctional CD8^+^ T cells that express the highest levels of IFN-γ are typically of the effector and effector memory (T_EM_) phenotype [44–46]. The majority of epitope mapping studies in immunogenicity have primarily used the IFN-γ ELISpot assay, which does not always correlate with control of viral replication or disease progression. T_CM_ responses, on the other hand, are thought to be more important for controlling HIV infection [47–49]. In the present study, we observed that all cytokine producing cells were memory T cells of either central memory or effector phenotype. These findings highlight the importance of developing a vaccine that can specifically induce polyfunctional T_EM_ or T_CM_ responses.

Earlier studies have reported that T cell responses were predominantly seen against Env and Gag peptides [26–27, 50], and that Env and Gag-specific responses had an inverse relationship with viral replication [51, 52]. In natural HIV infection, Env-specific CD8^+^ T cell responses have been shown to be associated with poor control of viral replication as compared to Gag-specific responses [53]. Nonhuman primates immunized with DNA plus Ad5 expressing SIV Env and Gag were found to be better protected against SIV challenge as compared to animals immunized with vaccines expressing only Gag [54]. These results suggest that generation of Env-specific responses might play an important role in early HIV infection, and show that a prime boost vaccination strategy elicits better cellular immune responses, predominantly against the Env and Gag antigens.

Memory T cell development and proliferation is associated with protective immunity. Hence, induction of memory T cells with high proliferative potential should be an important goal for vaccine development [55–57]. Local recruitment of cytolytic CD8^+^ T cells to the site of infection is critical for the elimination of infected cells and analysis of virus specific CD8^+^ T cells may provide critical information for the design of novel immunotherapies targeting HIV-infected CD4^+^ T cells [58–60]. Activation of naïve CD8^+^ T cells and their effective responses requires the clonal expansion and development of effector cells targeting the peptides of virally infected cells. This leads to the clearance of infected cells by cytokines and other immunologically active proteins such as perforins, granzymes, and chemokines such as MIP-1α/β and RANTES [61].

Significant polyfunctional T cell responses have been reported in HIV-1 infected long term non progressors (LTNP) [62], as well as in those vaccinated with Hepatitis B vaccine, HIV vaccine [63, 64], and vaccinia [65]. Studies have also shown that the frequency of HIV-specific polyfunctional T cells correlated inversely with viral load in progressors, clearly indicating a role for polyfunctional T cells in mediating protection [66]. Subsequent studies have shown that maintenance of highly functional CD4^+^ T cells co-producing IFN-γ, IL-2 and TNF- α in response to HIV-1 Gag, are necessary for suppression of viremia [67]. Studies that have looked at bi-functional T cells have also demonstrated correlation with protection against disease progression. A more recent study reported that chronically infected HIV-1 positive individuals on long-term antiretroviral therapy in combination with an HIV-1 virion-based therapeutic vaccine showed sustained augmentation of Gag/Pol-specific memory CTLs co-expressing IFN-γ and IL-2 that is generally associated with an effective immune response [68]. Similarly, HIV-1 Gag-specific CD4^+^ T cells secreting IFN-γ and IL-2 have been shown to correlate with protection in LTNP and elite controller phenotypes (EC) [69]. Studies have also demonstrated that HIV-2-infected individuals produce more functionally superior HIV-specific T cell responses characterized by highly polyfunctional HIV-specific CD4^+^ and CD8^+^ T cells [70]. Hence, multi-functional T cells constitute an important immune correlate of HIV vaccine efficacy.

Majority of the vaccine-induced polyfunctional, bi-functional, and mono functional CD8^+^ T cells were found be Env-specific. Group B volunteers had significantly more polyfunctional T cells than Group A volunteers (S4 Table), at two week post last MVA vaccination,(Gag-p = 0.004, Pol- p = 0.010, Env-p = 0.054).On the other hand, induction of Gag and Pol specific polyfunctional T cells was found to be sporadic in both the groups. The magnitude of the ICS response (bi functional IFN-γ TNF-α or IFN-γ IL-2 or IL-2 TNF-α and mono functional IFN-γ or IL-2 or TNF-α (Figs 5 and 6) (Tables 3 and 4) (S4 Table) was more predominant in group B than in group A.

There was a significant increase in Env-specific CD4^+^ T cells expressing TNF-α or IL-2 or co expressing TNF-α/IFN-γ (p = 0.004) and TNF-α/IL-2 (p = 0.005) post MVA boosting. Similarly, Env, Gag and Pol-specific CD8^+^ T cells expressing IFN-γ (Gag-p = 0.007, Pol-p = 0.004), TNF-α (Env-p = 0.037), IL-2 (Env-p = 0.007) or co expressing TNF-α/IFN-γ (Gag-p = 0.003, Pol-p = 0.003, Env-p = 0.046) and IFN-γ/IL-2 (Pol-p = 0.022) also increased significantly post MVA vaccination. The magnitude of the response was higher in vaccinees who received the heterologous regimen than those who received the homologous regimen. These results mirror the findings of the Phase I clinical trial conducted in London with the same vaccine, which reported that the vaccine successfully induced HIV-specific CD4^+^ T cells that predominantly secreted IL-2, IFN-γ, and TNF-α in response to Env stimulation. Our findings also showed that Env and Gag specific polyfunctional CD8^+^ and CD4^+^ T cells increased in both groups with boosting, although significance was observed in Group B only. The frequency bi functional, and mono functional CD8^+^ and CD4^+^ T cells was significantly increased in those who received the heterologous prime boost regimen.

We observed maximum induction of T cell immune responses at week 2 post final vaccination. The vaccine-specific cytokine response was significantly higher in Group A as compared to Group B, indicating that cell mediated immune responses were enhanced significantly by the DNA prime followed by the MVA boost. The difference in the number of doses of vaccine (4 for group A vs 3 for group B) could be one of the reasons for the observed difference between the groups. In contrast, certain other therapeutic HIV vaccine trials reported induction of polyfunctional CD4^+^ T cell response [71] and poly functional CD4^+^ and CD8^+^T cell response in volunteers who received a replication-defective HIV-1 vaccine (HIVAX) [72]. The current study showed that the CD4^+^ and CD8^+^ T cell responses were largely specific to Env antigens, followed by Gag and few for Pol and Nef-Tat.

Although cell mediated immune responses were not preferentially expanded in Group B participants, we reported in an earlier study that vaccination resulted in a strong induction of antibodies targeting the variable regions of HIV-1gp120, which correlated positively with induction of long-lasting plasma B cells and T Follicular Helper cells in volunteers who received only MVA [43]. Interesting observations of HIV-specific antibodies targeting the rgp41 Env, rgp140 Env, p24 Gag, and rgp120 Env were also made in the P002 trial conducted at UK with the same vaccine constructs [26]. This suggests that priming and boosting with the MVA vaccine can induce both cellular and humoral immune response. However, the very small sample size is a major limitation of the present study, which limits the power of the analytical comparison between the two groups

To summarize, the findings of the present study clearly demonstrate that priming and MVA boosting strategies effectively generate long-lived memory cells and induce activation of vaccine-induced anti-HIV-1 effector cells, thus encouraging us to propose that a subsequent trial in a larger population should be undertaken to provide further evidence to support the efficacy of the vaccine candidate and the vaccination strategy employed in this trial.

## Supporting information

S1 Fig Flow gating strategy for ICS. A time vs. CD3 APC H7 was first applied to ensure that acquisition of data occurred without blockages; following this a FSC-H vs. FSC-A gate was applied in order to exclude doublets and cell clumps. Once the lymphocyte population was identified, a dump gate (live dead stain) was applied to ensure that non-viable cells are excluded from analysis. The CD4^+^ and CD8^+^ T cells gates were applied in a similar manner, CCR7 and CD45RO was used to identify the generous CD4^+^ and CD8^+^ T memory cell gates for each cytokine. Each cytokine was gated vs. the opposite lineage and polyfunctional responses were assessed using the Boolean function of FlowJo. Env Specific cells secreting various cytokines is shown here as an example.
(TIF)

S1 Table Commercial reagents used for multicolor flow cytometry. (XLSX)

S2 Table Elispot assay peptide pools and sequence details. (XLSX)

S3 Table ICS assay peptide pools and sequence details. (XLSX)

S4 Table Vaccine-induced antigen-specific cytokine secreting CD4^+^ and CD8^+^ T cell frequencies during vaccination. (XLSX)
