# Supplementary figures and images for: Induction and maintenance of bi-functional (IFN-γ + IL-2+ and IL-2+ TNF-α+) T cell responses by DNA prime MVA boosted subtype C prophylactic vaccine tested in a Phase I trial in India
Source: PLoS One. 2019 Mar 28;14(3):e0213911. doi: 10.1371/journal.pone.0213911 (PMC6438518; doi:10.1371/journal.pone.0213911)

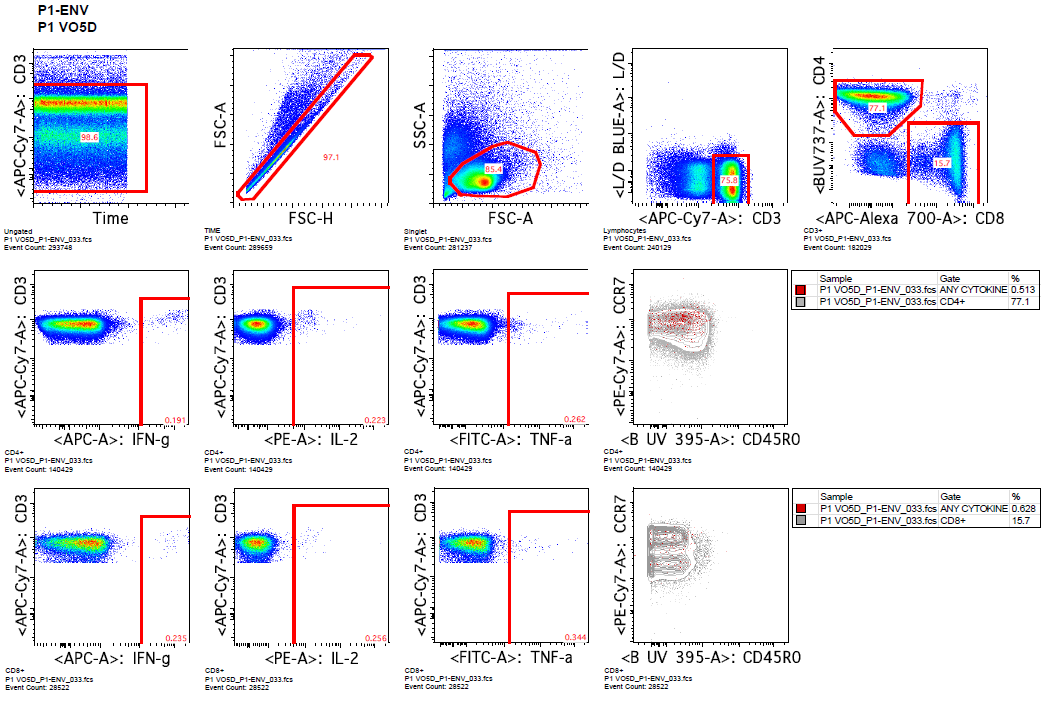

Supplement: S1 Fig — A time vs. CD3 APC H7 was first applied to ensure that acquisition of data occurred without blockages; following this a FSC-H vs. FSC-A gate was applied in order to exclude doublets and cell clumps. Once the lymphocyte population was identified, a dump gate (live dead stain) was applied to ensure that non-viable cells are excluded from analysis. The CD4+ and CD8+ T cells gates were applied in a similar manner, CCR7 and CD45RO was used to identify the generous CD4+ and CD8+ T memory cell gates for each cytokine. Each cytokine was gated vs. the opposite lineage and polyfunctional responses were assessed using the Boolean function of FlowJo. Env Specific cells secreting various cytokines is shown here as an example. (TIF) [file pone.0213911.s001.tif]
